# Temperature alters interactions and keystone taxa in the marine microbiome

**DOI:** 10.1093/ismejo/wraf287

**Published:** 2026-01-21

**Authors:** Ewa Merz, Riley J Hale, Erik Saberski, Kasia M Kenitz, Melissa L Carter, Jeff S Bowman, Andrew D Barton

**Affiliations:** Scripps Institution of Oceanography, University of California San Diego, La Jolla, CA 92037, United States; Scripps Institution of Oceanography, University of California San Diego, La Jolla, CA 92037, United States; Scripps Institution of Oceanography, University of California San Diego, La Jolla, CA 92037, United States; Scripps Institution of Oceanography, University of California San Diego, La Jolla, CA 92037, United States; Southern California Coastal Ocean Observing System, University of California San Diego, La Jolla, CA 92037, United States; Scripps Institution of Oceanography, University of California San Diego, La Jolla, CA 92037, United States; Southern California Coastal Ocean Observing System, University of California San Diego, La Jolla, CA 92037, United States; Scripps Institution of Oceanography, University of California San Diego, La Jolla, CA 92037, United States; Scripps Institution of Oceanography, University of California San Diego, La Jolla, CA 92037, United States; Department of Ecology, Behavior and Evolution, University of California San Diego, La Jolla, CA 92039, United States

**Keywords:** marine microbes, biotic interactions, keystone taxa, amplicon sequencing, empirical dynamic modelling

## Abstract

Marine microbes shape global biogeochemical cycles and marine food webs. Although biotic interactions underpin microbial community dynamics, most interactions between wild marine microbes are unknown. Here, we used empirical dynamic modeling to examine a six-year record of coastal microbial community composition to quantify microbial interactions and their changes through time. We found that, on average, marine microbes interact with 20% of other taxa in the community, most interactions are weak (80%), and that positive interactions are more common than negative interactions. Keystone taxa, defined as having disproportionally strong and frequent interactions, were not generally the most abundant taxa. The strength and sign of interactions, as well as the identity of the keystone taxa, varied through time and with changes in water temperature. An increase of 13°C, the dynamic range in water temperature at this location during the observational period, led to a 33% less interactive microbial community and an 11% shift towards more positive interactions. Only a few of the keystone taxa are the most interactive in the community at all times, and we found a temporal succession of keystone taxa. These results reveal that interactions in the marine microbiome are common, more facilitative than previously thought, and highly variable through time.

## Introduction

Biotic interactions shape microbial community composition, diversity, and temporal dynamics and, consequently, ecosystem functions [1–3]. Negative or inhibitory interactions, such as resource competition, predation, and parasitism, have long been known to regulate the spatial and temporal distribution of marine microbes [1, 4, 5]. Positive or facilitative interactions, such as the mutual exchange of nutrients and other small molecules, are also important [6–9]. For example, *Prochlorococcus*, the most abundant phytoplankton on Earth, grows in the presence of *Alteromonas* “helper” bacteria that metabolize their reactive oxygen species [10]. However, observing a broad range of interactions in a diverse microbial community in the wild has been challenging due to the scarcity of appropriate data and the lack of effective methods for identifying interactive relationships [11]. Consequently, our understanding of how wild marine microbes interact with each other remains quite limited outside of specific model systems.

Interactions in natural communities depend on the environmental context and may not be constant through time [12–14]. For example, competition between grazing zooplankton occurs only in periods of low planktivorous fish abundance [15]. In the case of *Prochlorococcus* and *Alteromonas*, the positive interaction breaks down at elevated carbon dioxide concentrations [16]. Our limited understanding of how microbial interactions change over time hinders our ability to predict how marine microbial communities will respond to environmental and human-induced climate change [17] and makes it difficult to mechanistically link changes in community composition to ecosystem functions, such as carbon uptake [18]. For example, *Prochlorococcus* contributes significantly to global net primary productivity, but its dynamics might not be accurately predicted without considering changes in interactions with their “helper” bacteria or the protists that prey on them [19].

Certain taxa in the community, known as keystone taxa, are more highly interactive than others, and a change in their abundance disproportionately affects ecosystem structure and function [20, 21]. Even though keystone taxa have been identified in various ecosystems [20, 22, 23] and microbiomes [24, 25], the presence, dynamics, and impacts of marine microbial keystone taxa remain poorly understood. Due to their importance, keystone taxa often serve as focal points in monitoring and conservation efforts, and the lack of knowledge of keystone taxa in the marine microbiome represents a significant gap in understanding and conserving these ecosystems.

Here, we paired a high-frequency, molecular microbial community composition time series with an empirical modeling approach to: (i) quantify the strength, sign, and frequency of marine microbial interactions and how they vary through time, (ii) identify keystone microbial taxa and how their interactions differ from other taxa, and (iii) determine whether interactions and keystone taxa are influenced by water temperature.

**Figure 1:**
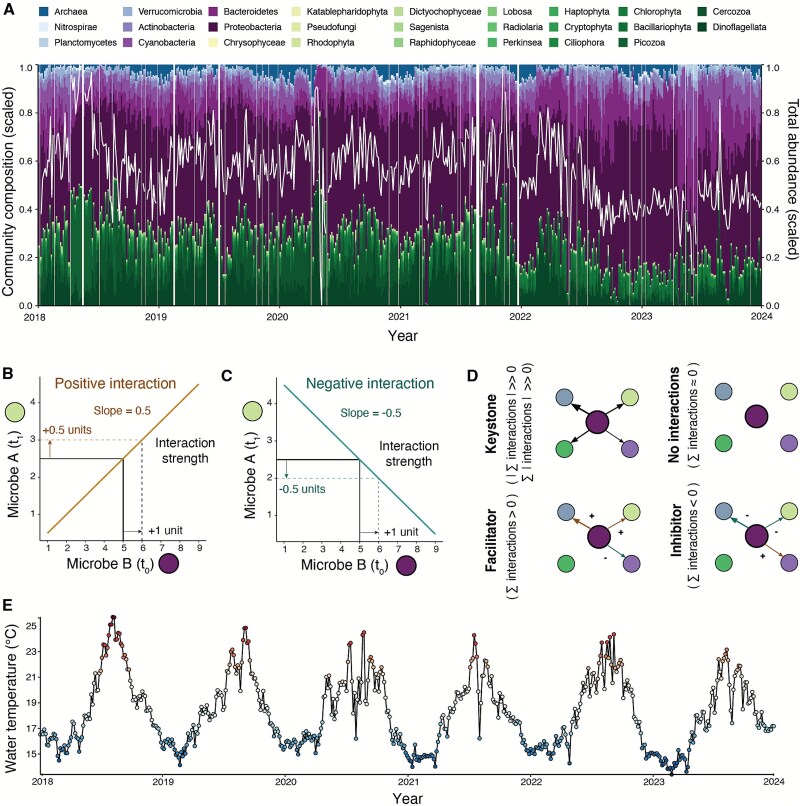
Six years of 16S and 18S amplicon sequencing and water temperature measurements to study interactions among microbes in a coastal environment. (A) Microbial community composition and total abundance. Only dominant taxa (average relative abundance over time > 0.1% and present >50% of samples) are displayed and included in the study. The white line shows fluctuations in the total relative abundance of dominant taxa (scaled). (B) Illustration of the meaning of positive interaction strength and (C) negative interaction strength for a point in time. (D) Interaction types. Colors correspond to higher taxonomic groups defined in (A). (E) Surface water temperature measured at Scripps pier between 2018 and 2024 in °C.

We estimated the relative abundance of marine microbes, here including Bacteria, Archaea, and Eukaryotes but not viruses, using six years of twice-weekly 16S and 18S rRNA gene amplicon sequencing data collected at the Scripps Pier in San Diego, California (Fig. 1A). Amplicon sequencing resolves species diversity across a broad range of sizes and taxonomic groups as well as intraspecific diversity [26, 27]. Several studies have explored interactions between uncultured microbes using time series of amplicon sequencing variants (ASVs). Some of these studies stand out due to their long durations, spanning many years in some cases, or their high temporal resolution [11, 25, 28, 29]. Our Scripps Pier time series and inferences about marine microbial interactions build upon these previous studies by sampling prokaryotic and eukaryotic microbial communities at high frequency for many years, with few missing values.

We used multiview distance regularized (MDR) S-maps, a non-parametric method based on state-space reconstruction, which is part of the Empirical Dynamic Modeling (EDM) framework [15, 25, 30]. This approach allowed us to infer time-varying interactions among the most common microbial ASVs, classified to the lowest possible taxonomic resolution. The MDR S-map has been used to effectively infer interactions using relative abundance data, though with slightly decreased accuracy [25] (Fig. S1). The inferred interactions are time-varying and indicate how changes in one microbe's abundance will affect another's abundance (Fig. S2, Fig. 1B–C, **Supplementary Materials and Methods**). Interactions were classified as positive (facilitative), negative (inhibitive), or neutral (i.e. indistinguishable from zero). A positive interaction means that an increase in the abundance of one microbe will result in an increase in the abundance of another microbe in the next time step. Conversely, a negative interaction will lead to a decrease in the abundance of the other microbe. We defined keystone taxa as microbes that interacted strongly with other microbes and had a high net effect on the entire community (Fig. 1D). To produce the most robust results for the most relevant microbes, we only considered the 162 microbial taxa with a mean relative abundance greater than 0.1% that occur in at least 50% of the samples. We also included surface water temperature, which has both direct and indirect effects on microbial communities and is correlated with other environmental variables [5], in our MDR S-map models to isolate microbial interactions from environmental drivers (**Methods**, Fig. 1E).

The MDR S-map method provides valuable estimates of the strength and direction of interactions between microbes, but does not reveal the underlying mechanisms behind these interactions. For example, a negative interaction may result from competition, predation, or the production of toxins. Although alternative methods exist for assessing interactions, such as controlled laboratory experiments [1] or co-occurrence networks derived from field observations [28, 29], the use of high-throughput molecular data and non-parametric methods offers significant advantages. Our approach enables the study of multiple simultaneously interacting microbes in their natural environments, taking into account their nonlinear dynamics and how these interactions change over time. The improved taxa- and community-level view of interactions has implications for understanding and predictions of microbial community structure and dynamics, marine food webs, and global biogeochemical cycles.

## Materials and methods

### Data collection

#### Microbial community structure

We collected data on microbial community structure twice weekly (nominally Monday and Thursday morning) for 6 years (2018–2023) from the Ellen Browning Scripps Pier at the Scripps Institution of Oceanography (SIO) in San Diego, CA (Fig. 1A). This sampling was part of two ongoing projects: The Scripps Ecological Observatory (www.ecoobs.ucds.edu) and the Southern California Coastal Ocean Observing System Harmful Algal Bloom Monitoring and Alert program at Scripps Pier (SCCOOS HABMAP, https://sccoos.org/harmful-algal-bloom/).

Amplifying and sequencing 16S and 18S rRNA genes is a standard procedure to determine marine microbial community structure. We used 16S and 18S rRNA gene amplicon sequencing data from the Scripps Pier as described in a previous study [26], adding three additional years by following the same procedure. Briefly, surface seawater (1 liter) was filtered through a sterile 0.2 μm Supor membrane disc filter (Pall Corporation, Port Washington, NY, USA) and stored at −80°C until extraction. We then manually extracted DNA with the MoBio DNEASY PowerWater Kit (Qiagen, Venlo, Netherlands) during the first year (2018) the KingFisher Flex Purification System and MagMax Microbiome Ultra Nucleic Acid Extraction Kit (ThermoFisher Scientific, Waltham, United States) in subsequent years. The Argonne National Laboratory Environmental Sample Preparation and Sequencing Facility amplified and sequenced extracted DNA using the MiSeq platform (Illumina), based on a 2 × 151 bp library architecture using the 16S V4 primers 515F and 806R for bacteria and archaea [31]. We used the software DADA2 [32] to filter, trim, denoise, and merge the obtained 16S and 18S rRNA sequences as ASVs. Chimeras were removed with removeBimeraDenovo in DADA2. To all ASVs we assigned the lowest possible taxonomic classification (internal branches or terminal branches on the reference tree) using the software paprica v0.7.0 (https://github.com/bowmanjeffs/paprica) [33] with a secondary classification using the Bayesian classifier in DADA2 against Silva v138.1 [34]. Paprica uses RefSeq as a reference database (https://www.ncbi.nlm.nih.gov/refseq/) and assigns taxonomy using a phylogenetic placement approach, where each ASV is placed onto a pre-computed phylogenetic tree with Infernal [35], epa-ng [36], and Gappa [37]. ASVs that were classified as chloroplasts or mitochondria were removed from further analysis. We ensured consistency throughout the time series by examining the widespread recurrence of ASVs. Trimming parameters (using filtrerAndTrim in DADA2 with the flags trimLeft = 15 and truncLen = 150 for 16S and default settings for 18S) were standardized across the time series, and quality plots were manually inspected for each run before and after quality control to ensure adequate trimming and filtering of reads. The amplicon sequencing libraries for this project is available on NCBI SRA (BioProject PRJNA662174).

#### Water temperature

We used daily surface water temperature measurements from the Scripps Pier Shore Stations Program, as described in a previously published reference [38] (Fig. 1D). Staff from the Scripps Institution of Oceanography, Birch aquarists, and volunteers took daily temperature readings at the sea surface from the end of Scripps Pier.

### Estimation and analysis of interactions

#### Multiview distance regularized S-maps to infer interactions from time series

We used the MDR S-map from the EDM framework to estimate time-varying interaction strengths and directions among marine microbes [25]. S-maps are locally weighted multiple linear regressions, where points closer in state-space reconstructions (SSRs) receive a higher weight [39]. To estimate interactions at a specific time point, we used the MDR S-map to predict the relative abundance of one ASV at the next time step based on its current relative abundance as well as that of all other ASVs. The MDR S-map’s coefficients approximate the Jacobian interaction matrix [15]. In our case, they describe how the changes in one ASV’s relative abundance will result in changes in another ASV’s relative abundance (Fig. 1B–C, **Supplementary**). We simplified the original MDR S-map approach, as described in the **Supplementary Materials and Methods,** to make it conceptually easier and computationally less intensive while maintaining the same efficacy in inferring interaction strengths [25].

Recently added regularization schemes make the S-map less prone to observational noise and collinearity among model parameters [30]. By averaging distances among points across multiple smaller SSRs, each smaller than the community size (multiview distance), MDR S-maps can reduce overfitting and reconstruct interaction networks of *n*-dimensional communities [25]. We divided the MDR S-map into three steps: (i) calculate the distance between the sampling points, (ii) parametrize the local regularized regression, and (iii) extract the local model coefficient. We provide a detailed description of those steps and how we applied them to our data in the **Supplementary Materials and Methods**.

Before using the MDR S-map, we selected only the most common ASVs, binned the data over 4 days, and standardized the time series to have a zero mean and a standard deviation of 1. We focused on the most common ASVs because rare ASVs (with many zero relative abundances) are unsuitable for time series analysis [12], and we aimed to focus our study on the most prevalent microbial interactions. We selected the most common ASVs based on two criteria used by a previous study [25]: (i) their relative abundance, when present, was above a detection threshold of 0.1%, and (ii) they occurred in at least 50% of observations over time (relative abundance >0). This procedure yielded 186 ASVs (out of a total of 53 083 ASVs), with 133 ASVs originating from 16S rRNA gene sequencing and 72 from 18S rRNA gene sequencing. More details (e.g. taxonomic classification) on the dominant ASVs are available on GitHub (https://github.com/ew-merz/microbial-interactions) and Zenodo (10.5281/zenodo.17595082). Although the reduction in total ASVs resulting from these criteria is extreme, it ensured that our estimates of interactions among the remaining taxa were robust. After selecting only common ASVs, we aggregated the remaining data into discrete bins over 4 days to ensure a fixed sampling interval [40]. Bins of 4 days yielded the maximum number of preserved data points for analysis (Fig. S3). Finally, we standardized the ASV time series for a zero mean and a standard deviation of 1 to ensure that all ASVs had the same level of magnitude for comparison and to avoid distorted state-space reconstructions [40].

We included surface water temperature as a covariate in the MDR S-map models to account for correlations among ASV abundances in response to a common strong driver. We chose temperature because it indirectly accounts for various other environmental covariates, such as nutrient availability or upwelling. In other words, this step gave more confidence that the estimated interaction strengths were due to taxa-taxa effects rather than the environment. We discuss other steps to increase confidence in our inferred interaction strengths in the **Supplementary Materials and Methods** section and show that seasonal signals do not influence the inferred interaction strengths (Fig. S4).

#### Strength, direction, and frequency of marine microbial interactions

**Figure 2:**
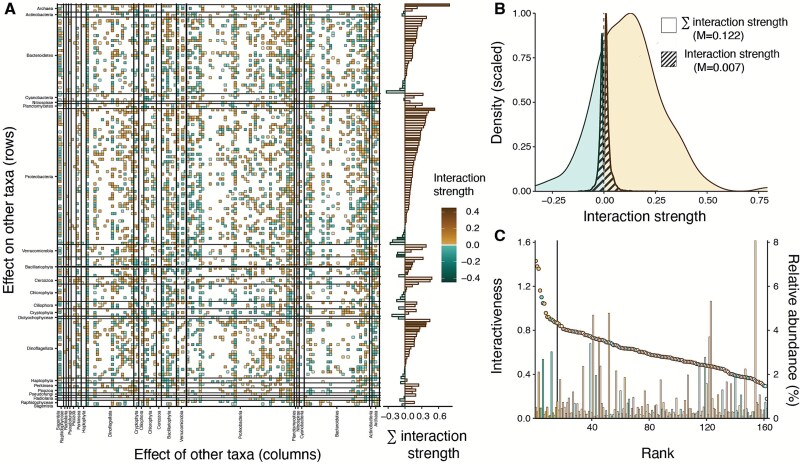
Marine microbes are highly interactive, with predominantly facilitative interactions. (A) Average interactions over time (matrix on the left) and the sum of interaction strengths across interactive partners for each taxon (bar plot on the right). Examining the rows reveals how different taxa influence those shown in the column of the interaction matrix. The shaded regions show the 10% most interactive microbes. (B) Kernel density estimation of all non-zero interaction strengths within the community for all taxa (cross-hatched) and the sum of interaction strength for each taxon (non-cross-hatched). M stands for the median of the distribution. (C) Interactiveness and relative abundance. Interactiveness is defined as ${\left({\left(\varSigma\ interaction\ strength\right)}^2+{\left(\varSigma | interaction\ strength|\right)}^2\right)}^{0.5}$. Dots show the interactiveness, whereas bars show the relative abundance of each microbe. The black vertical line (located at rank 16) indicates the top 10% of the most interactive microbes, referred to as keystone taxa. Colors in (B) and (C) correspond to the direction of interaction strength defined in (A).

We only considered ASVs with a clear taxonomic classification (e.g. excluding “phylum reps”) for our analysis, which resulted in 162 microbial ASVs. We restricted all our analyses to strong interactions to reduce the influence of noise and false positives. We defined strong interactions as those having a strength greater than 0.005 and occurring more than 5.7% of the time. The thresholds for strength and occurrence were determined based on the upper 50% quantile of their respective distributions. The strongest pairwise interactions were averaged over time (Fig. 2A). We calculated the net interaction strength by summing the time-averaged interactions over all taxa (Fig. 2A). The $\varSigma\ interaction\ strength$ shows the taxon’s net interactive effect on the community because negative and positive interactions can cancel each other. The $\varSigma \mid interaction\ strength\mid )$, in contrast, shows the absolute strength of pairwise interactions becausee negative and positive interactions do not cancel each other. To determine the strength, direction, and frequency of microbial interactions, we plotted the density distribution of the net interaction strength and pairwise interaction strengths (Fig. 2B). Interactiveness (Fig. 2C) was calculated as ${\left({\left(\varSigma\ interaction\ strength\right)}^2+{\left(\varSigma \left| interaction\ strength\right|\right)}^2\right)}^{0.5}$and thus included both net interaction strength and net absolute strength of pairwise interactions.

#### Identifying and characterizing keystone microbes

**Figure 3:**
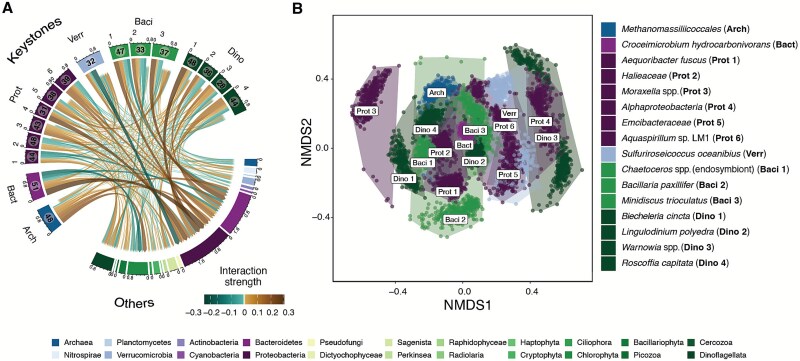
Keystone microbial interactions. (A) Chord diagram of keystone microbial interactions. Chord widths correspond to the net interaction strength averaged over time. Chord colors indicate the strength and sign of the interaction. Numbers on the dial show the total number of interactions with other microbes (links). The colors of chords show the direction of interactions, where green means negative and brown is positive. (B) NMDS analysis of keystone microbial interactions. Dots display a single time point, whereas shapes are polygons containing all points of a given keystone microbe. We excluded all points with a stress value greater than 0.2.

We defined the 10% most interactive microbes (highest interactiveness) as keystone taxa (Fig. 2C and Fig. 3). We selected them based on interactiveness because they had the highest net interaction strength and highest net pairwise interaction strength.

We utilized non-metric multidimensional scaling (NMDS) to investigate the interactions among keystone microbes by comparing the identity and strength of these interactions at each time point [41]. Unlike principal component analysis (PCA), NMDS does not assume linearity, thereby accommodating various data types, including non-normal data, missing values, and null entries. NMDS focuses on maximizing the rank order between samples and can apply any dissimilarity measure for comparison. For our analysis, we employed the Bray–Curtis dissimilarity metric.

#### Context-dependency of microbial interactions

**Figure 4:**
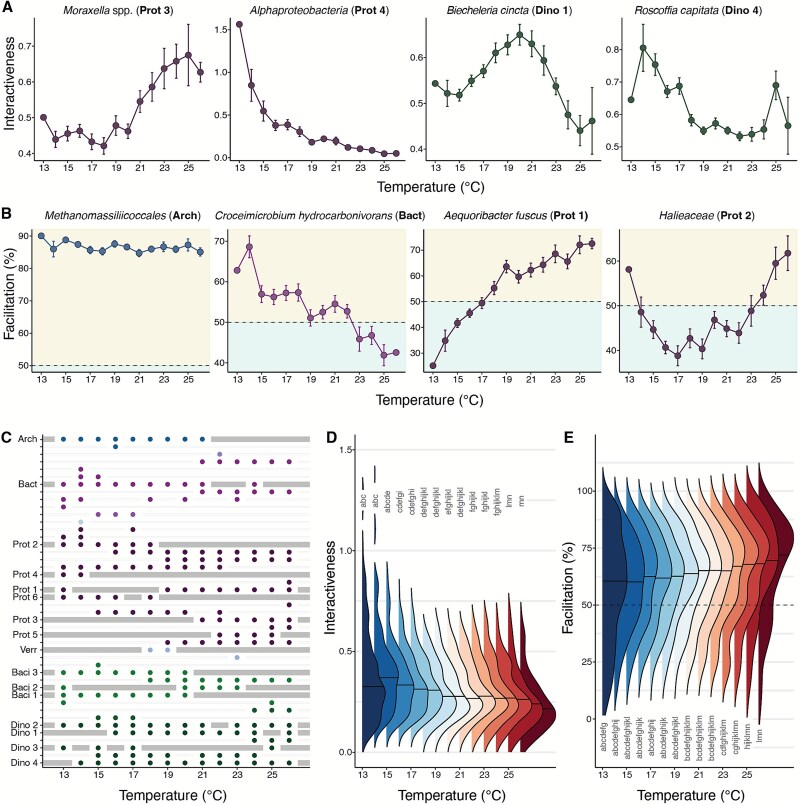
Microbial interactions change with the environment. Temperature dependency of (A) interactiveness and (B) percent facilitation of selected keystone taxa. Percent facilitation is calculated by dividing the sum of positive interactions by the total absolute strength of interactions multiplied by 100 at a given temperature. The shading denotes the net positive (facilitation >50%) versus net negative interactions (facilitation <50%). Error bars in (A, B) show the standard error of the mean (SE) using all interaction data from a given temperature. Colors of dots and lines in (A, B) correspond to higher taxonomic groups shown in Fig. 1A. (C) Turnover in keystone microbes as a function of temperature. Dots indicate that a particular taxon is a keystone for a specific temperature, whereas bars signify that a keystone mentioned in Fig. 3 would not be classified as a keystone for that temperature. Abbreviations in (A–C) correspond to the keystones in Fig. 3. Density distributions of interactiveness (D) and (E) percent facilitation in the whole community as a function of water temperature. If the distributions do not contain the same letter, they are significantly different from each other (Kolmogorov–Smirnov test, P value <0.05; see also Fig. S14).

To investigate if microbial interactions changed with temperature, we examined the interactiveness and percentage of keystone microbes' positive interactions (facilitation %) along a temperature gradient (Fig. 4A–B). Facilitation is the sum of the strength of the positive interactions divided by the total absolute strength of interactions multiplied by 100, or $\left(\varSigma\ interaction\ strength>0\right)/\left(\varSigma | interaction\ strength|\right)\times 100$). The temperature gradient consisted of 14 bins, ranging from 13°C to 26°C, and we sorted the temperature data into the bins by rounding each observed temperature value to 0 digits (e.g. a temperature value of 17.2°C would be sorted into the bin of 17°C). Moreover, we examined how the keystone microbial community would change depending on temperature by selecting the top 10% most interactive microbes for each temperature level (Fig. 4C). To study community-level effects, we studied the density distributions of interactiveness and facilitation % along the temperature gradient (Fig. 4D-E).

## Results and discussion

### Marine microbes are highly interactive but most interactions are weak

We found that even though marine microbes had many interactive partners in the community, most interactions were weak. We averaged the interaction strengths over time to estimate the average interaction strength of each microbe with others (Fig. 2A, Fig. S5). Of the 162 best-sampled microbial taxa considered in our analysis, each interacted with 20% of the others, on average. The median strength of interactions across all taxa was relatively weak (median = 0.007; Fig. 2B). The predominance of weak interactions in the community has been observed for other groups of organisms and ecosystems [42, 43]. Because weak interactions within a diverse ecological community promote persistence and stability [44–47], this observed property of the coastal marine microbiome may be critically important for regulating microbial community change through time.

The number, strength, and direction of interactions varies across major taxonomic groups. Members of the phylum Actinobacteria had the most interactions with others (37.5 of 162 possible interactions on average in this group), followed by Cercozoa (36) and Planctomycetes (36). Because most interactions changed over time, not all possible interactions are realized at each time point. Cercozoans, key predators of bacteria, were the most interconnected taxonomic group over time. Approximately 53.5% of the time, their possible interactions with other taxa were different from zero. Among the different groups, Archaea had the strongest interactions with other microbes, with an average interaction strength of 0.11. Furthermore, Archaea had the highest number of facilitative (positive) interactions, whereas Dictyochophyceae and Bacillariophyta showed the most inhibiting (negative) interactions. Archaea are ubiquitous in marine environments and mediate global carbon and nutrient cycles, such as nitrogen, iron, and the degradation of organic matter [48]. Diatoms (Bacillariophyta) are fast-growing, opportunistic phytoplankton that often form dense blooms in nutrient-rich conditions [49]. The causes of these differences in interactions are unclear, but they are likely to have important implications because major taxonomic groups play distinct roles in food webs and biogeochemical cycling, e.g. including the drawdown of silica [50] and efficient upward trophic transfer by diatoms [51].

### Positive interactions are more common than negative interactions

Positive interactions, or facilitation, were more common than negative interactions among marine microbes. We examined the signs of interactions from two perspectives. First, we examined the character of interactions between all possible pairs of microbes, providing a broad sense of whether interactions were more positive or negative in the community. Second, we considered the net interaction for each taxon when summing the interaction strength across microbes ($\varSigma\ interaction\ strength$); we call this latter metric net interaction strength. Of possible pairwise interactions across the community (Fig. 2A, Fig. S5), 11.1% were positive, 8.8% negative, and 80.1% neutral (i.e. indistinguishable from zero). Pairwise interaction strengths were right-skewed toward positive interactions, showing a non-normal distribution (skewness = 0.402; Jarque-Bera Normality Test *P* value = 0.0002992; Fig. 2B). This indicates that, overall, positive pairwise interactions outnumber negative ones in the community. Cercozoans had the most substantial pairwise interaction strength, exceeding the average across major taxonomic groups by more than double (Fig. 2A; pairwise strength_average_ = 0.121, pairwise strength_Cercozoa_ = 0.388). This group is an important component of the microbial loop because they consume bacteria and serve as food for other organisms, such as small invertebrates, fish larvae, and other zooplankton [52]. Most microbes displayed an overall positive net interaction strength with other microbes, with 78% exhibiting positive net interactions (n_positive_ = 125, n_negative_ = 36). Furthermore, positive net interaction strengths were considerably stronger than negative ones (strength_positive_ = 0.179, strength_negative_ = −0.081). Net interaction strengths were right-skewed toward positive interactions, showing a non-normal distribution (skewness = 0.820; Jarque-Bera Normality Test; *P* value <2.2e-16; Fig. 2B).

The dominance of positive interactions was unexpected, given the evidence for and focus on negative interactions among marine microbes, including but not limited to predation, competition for resources, and toxin production [1, 53–56]. However, in support of our conclusion, two recent studies have found more positive than negative interactions in microbial communities [25, 29]. Many examples of facilitative interactions exist within marine microbial communities that could explain the ubiquity of positive interactions, including cross-feeding and codependency, nitrogen fixation, facilitated succession on particles, direct exchanges of metabolites through nanotubes, and the degradation of dead organic material [7, 8, 57–59]. To date, positive interactions between species are not yet explicitly represented in state-of-the-art predictive models of microbial communities [60, 61], despite their abundance in observed microbial communities and importance for promoting community stability [25].

The stress gradient hypothesis states that positive interactions, or facilitation, increase in more stressful environments, where stress may include a range of biotic or abiotic factors [6, 62, 63]. In the coastal marine environment in Southern California, macronutrients such as nitrogen are often very low, limiting microbial growth and net primary productivity, and shaping microbial community composition [5]. Though evidence for the stress gradient hypothesis in aquatic microbial communities is limited, a study showed that rates of facilitation between microbes were higher in harsher conditions in industrial waste fluids [64]. The dominance of positive interactions in the coastal marine microbiome (Fig. 2) may be driven by persistent stress in this habitat and lend support to the stress gradient hypothesis.

### Highly interactive keystone microbial taxa

Certain microbial taxa exhibited considerably more interactions than others, but these taxa were not generally the most abundant in the community. Our findings indicated that the net interaction that a microbe has on the community displayed more extreme values than what would be expected from a normal distribution (unfilled distribution in Fig. 2B, kurtosis = 4.32). In other words, a limited number of microbial taxa were exceptionally interactive. To identify the most interactive microbes, we calculated an additional metric that we called “interactiveness,” which combined the net interaction across the community and the absolute strength of pairwise interactions ${\left({\left(\varSigma\ interaction\ strength\right)}^2+{\left(\varSigma \left| interaction\ strength\right|\right)}^2\right)}^{0.5}$. Although numerous metrics are available to define keystone taxa based on interaction networks (e.g. centrality; [65]), we opted to develop our own simple, composite metric that measures the overall interaction strength of a microbe within the community and the individual strength of its pairwise interactions. We compared the interactiveness of each microbe with its abundance. We found that the most interactive microbes were infrequently the most abundant ones (Fig. 2C, Fig. S6). For example, the most abundant ASV (8.2% average relative abundance) was a *Candidatus* Pelagibacter spp. of the SAR11 clade (Fig. S7). However, this ASV ranked only 158th out of 162 in terms of interactiveness.

Keystone microbial taxa were defined as taxa that interacted more frequently and strongly than non-keystone taxa, with their interactions often being either more positive or more negative than average. Here, we defined keystone taxa as the top 10% of the most interactive microbes based on their interactiveness (Fig. 2C, Fig. 3). These 16 keystone taxa out of 162 microbes accounted for 17% of all interactions and exerted a strong influence on other community members. Additionally, keystone microbes, by definition, tend to have more extreme negative or positive net interaction strengths than non-keystone taxa. Like other terrestrial and aquatic communities, keystone microbial taxa can strongly shape marine microbial community composition [20, 22, 23]. Because of their critical role in shaping community composition and dynamics, these keystone taxa are also likely to regulate ecosystem functions, such as carbon uptake and export from the surface [21, 24]. However, this study did not explicitly test whether keystone taxa regulated ecosystem functions, which represents a limitation in identifying keystones based solely on their interactiveness [24].

The keystone microbes we identified primarily belonged to three groups: Proteobacteria (6 ASVs), Dinoflagellates (4 ASVs), and Bacillariophyta (diatoms) (3 ASVs) (Fig. 3A). One of the dinoflagellate ASVs belonged to *Lingulodinium polyedra*, a harmful algal taxon that blooms strongly and frequently in the Southern California Bight [66]. For example, in spring 2020, a historically strong bloom of *L. polyedra* greatly reduced surface and subsurface oxygen concentrations in estuaries and coastal zones, and fish and invertebrate stranding and mortality were observed [67]. Previous work showed a strong change in bacterial community composition in response to the *L. polyedra* bloom, driven by rapidly fluctuating oxygen levels and the abundance of dinoflagellate-derived photosynthate [27]. Although the ecological role of some keystone phytoplankton like *L. polyedra* is well-studied, the functions of other taxa are less clearly identified due to insufficient observational data and the difficulty of culturing them in laboratory settings.

### Interactive networks for keystone taxa differ from those of non-keystone taxa

Keystone microbial interactions showed greater consistency over time than other microbes, sometimes involving distinct partners that contributed to their unique interactive roles in the community. We used non-metric multidimensional scaling (NMDS) to explore the similarity of interactions among keystone taxa using interaction strengths calculated for each time point. Keystone microbial interactions were more consistent over time and distinct from one another compared to other microbes. This was depicted by a shorter distance between points in an NMDS plot within the same taxa and a greater distance between different taxa, compared to an NMDS plot done with non-keystone microbes (Fig. 3B, Figs. S8 and S9). In addition, some keystone taxa had interactive networks that clustered more densely than others, indicating that their interactions were quite similar to each other. This was particularly true for all members of Bacillariophyta. In contrast, other keystone taxa had interactive networks that did not overlap with other taxa in the NMDS plot. For example, *Moraxella* spp., *Alphaproteobacteria*, and *Warnowia* spp*.*, which belong to the Proteobacteria and Dinoflagellate groups, displayed distinct interactions. The emerging view based on these data is that keystone taxa are not only the most interactive but also tend to have interactive networks more distinct than average compared to other taxa. The physical and biotic mechanisms shaping this pattern are unknown. However, we hypothesize that microbes with distinctive interactions, such as *Warnowia* spp., are particularly important to their community because their ecological impacts may be difficult to replace if they become rare.

### Water temperature affects microbial interactions and keystone taxa

The strength and sign of interactions among the taxa are not constant through time and change with temperature. To explore the environmental dependency of interactions, we averaged the level of interactiveness and the percent of facilitative interactions (defined as the sum of the strength of the positive interactions divided by the total absolute strength of interactions multiplied by 100, or $\left(\varSigma\ interaction\ strength>0\right)/\left(\varSigma | interaction\ strength|\right)\times 100$) for each microbe across the observed environmental temperature gradient at Scripps Pier (2018–2023; 13°C–27°C, with a temperature range of 14°C). We focused on temperature rather than other environmental variables because it directly and indirectly affects microbial physiology, interactions, and community composition [68–70]. Because temperature influences water column stratification and other oceanographic processes, it often covaries with nutrients, light, and other influential environmental factors.

We examined how the interactions of keystone taxa and the community as a whole changed across the gradient in temperature, and found that inter-taxa interactions often, but not always, depend upon temperature. We illustrate this by considering just keystone taxa. Whereas some keystone taxa became more interactive or facilitative as temperatures increased, others exhibited the opposite pattern (Fig. 4A–B, Fig. S10-Fig. S11). For instance, the interactiveness of the proteobacterium *Moraxella* spp. increased with higher temperatures, whereas the interactiveness of *Alphaproteobacteria* declined with higher temperatures. Other keystone taxa, such as the dinoflagellate *Biecheleria cincta* and *Roscoffia capitata*, showed the highest interactiveness at intermediate and extreme temperatures, respectively. One possible explanation for the observed interactiveness at varying temperature levels could be a positive correlation between the abundance and interactiveness of keystone taxa. At certain temperature levels, these taxa may interact strongly because they are more abundant. We found a positive correlation between increases in relative abundance and interactiveness as a function of temperature for only seven out of the 16 keystone taxa (Fig. S12). This may account for some, but not all, of the observed patterns. Some keystone microbes, like *Croceimicrobium hydrocarbonovorans*, became more inhibitory as temperatures increased, whereas others, such as *Aequoribacter fuscus*, became more facilitative. Certain microbes, like *Halieacea*, only inhibited others at intermediate temperatures. Others, such as *Methanomassillicoccales*, consistently facilitated the growth of other microbes, regardless of temperature. Many microbial traits, such as growth and metabolic rates, as well as interactions like grazing, scale with temperature [71]. Consequently, species fitness varies across temperature [49]. Furthermore, increasing evidence suggests that interactions among taxa may vary depending on environmental context [15, 16, 25].

The taxa identified as keystones varied with temperature, highlighting the dynamic nature of microbial communities. To demonstrate this, we selected the 10% most interactive microbes for each temperature along a gradient. Our findings indicated that some keystone microbes, like Methanomassillicoccales, were only among the most interactive in cold environments, although others, such as *Aequoribacter fuscus*, were among the most interactive in warm environments (Fig. 4C). Furthermore, we identified several microbes that were keystones within a restricted temperature range, but their overall interactiveness was insufficient to rank among the top 10% most interactive across all temperatures. This subset included *Candidatus* Pelagibacter spp. (FZCC0015) and *Candidatus* Endolissoclinum faulkneri, which belonged to the Proteobacteria phylum. When examining this temperature-dependent set of keystone taxa over the entire studied period, we observed a temporal change in their interactiveness (Fig. S13). In other words, there is a succession of keystone taxa through time. The ocean undergoes significant environmental changes across various timescales, ranging from daily fluctuations to changes that occur over centuries or longer [72]. As a result, the most influential keystone taxa within these communities and their substantial effects on community dynamics and ecosystem functions are likely to shift over time. In many ecosystems, the taxa thought to act as keystone species are considered relatively stable over human observational timescales, barring extinction or extirpation. In contrast, marine microbes have very short generation times and experience tremendous change in community composition over weeks to months [5, 26, 73, 74]. As the environment changes over the course of a year, the keystone microbial taxa also change.

Marine microbes interacted more in colder environments, whereas they were more facilitative in warmer environments when nutrient levels were typically low at this location. To examine the community-level effects of water temperature on microbial interactions, we studied distributions of interactiveness and the percentage of facilitation for each microbe along a temperature gradient. In colder environments, microbes demonstrated more extreme interactions (i.e. strongly positive or negative), as indicated by a flatter distribution with longer tails. Moreover, interactiveness overall was higher in colder environments (Median_13°C_ = 0.33, Median_26°C_ = 0.22, Fig. 4D, Fig. S14A). The long tails in the distribution of interactiveness (interactiveness greater than 1) indicate some hubs of highly interactive taxa at cold temperatures (13–14°C). Those seven highly interactive ASVs belong to the groups of Proteobacteria, Bacteroidetes, and Bacillariophyta, and six out of them had, on average, a positive interaction with other microbes. Facilitation occurred even more frequently in warmer environments (Median_13°C_ = 60.5%, Median_26°C_ = 72%, Fig. 4E, Fig. S14B), where nutrient levels are typically low at this location [75]. We speculate that the high interactiveness in cold, nutrient-rich conditions may be due to phytoplankton blooms, which tend to be dominated by a limited number of highly competitive species [66] that can directly influence the rest of the community (e.g. through competition and predation) or indirectly (e.g. through shading or the production of metabolites). The shift toward more facilitation at higher temperatures and lower nutrient conditions is consistent with the stress gradient hypothesis [6]. Challenging physical conditions, such as nutrient-depleted environments or high temperatures, can be alleviated by cooperation with organisms (e.g. cross-feeding), which may help explain the shift to greater facilitation in warmer conditions [6, 76]. These results suggest that as ocean temperatures change due to both natural and human processes, the levels of interactiveness and facilitation also change. This has implications not only for community composition but potentially also for community stability and resilience; e.g. the number of interactions can increase a community's robustness to species extinctions [77–80].

## Conclusion

Using high-throughput molecular time series data and an empirical modeling approach, we offer insights into how marine microbes interact with other taxa within their natural environment. Our findings revealed that marine microbes were highly interactive, with facilitation occurring more frequently than previously recognized. Whereas some of these interactions may have been studied and documented in the past in field and laboratory studies, many remain poorly understood [11, 25]. Additionally, we identified keystone taxa, defined as microbes that interacted disproportionately more than other taxa.

We also discovered that microbial interactions and the composition of keystone communities vary depending on the environmental context; e.g. they became less interactive but more facilitative at higher temperatures. Overall, we argue that understanding and predicting community dynamics within the marine microbiome depends upon detailed and still-emerging knowledge of inter- and intra-species interactions, in addition to bottom-up environmental changes and top-down predation processes [81].

Microbial community models are utilized to study species biogeography, community diversity, and biogeochemistry and to predict how ocean ecosystems respond to anthropogenic climate change [81–83]. Even though these models typically account for a few to tens of species, and interactions such as competition and predation, they often overlook facilitative interactions or other direct and indirect interactive processes. This is likely because, to date, we lack robust observations of these positive interactions in the wild or theoretical and mathematical descriptions of such interactions. We recommend that future microbial community models attempt to resolve various microbial interactions and leverage emerging field (such as ours) and laboratory studies documenting these interactions [84]. Critically, the strength and sign of interactions should vary with environmental conditions. A new generation of ecological models that resolves a fuller range of environmentally dependent interactions may facilitate more skillful and mechanistic predictions of ecosystem and biogeochemical dynamics in a changing environment.

## Supplementary Material

supplementary_wraf287(1)

## Data Availability

The data and code needed to reproduce the results presented in this manuscript are available on GitHub (https://github.com/ew-merz/microbial-interactions) and Zenodo (10.5281/zenodo.17595082).
