# Supplementary material for: Temperature alters interactions and keystone taxa in the marine microbiome
Source: ISME J. 2026 Jan 21;20(1):wraf287. doi: 10.1093/ismejo/wraf287 (PMC12822067; doi:10.1093/ismejo/wraf287)
Supplement: supplementary_wraf287(1) [file supplementary_wraf287(1).pdf]

Supporting Information for

Temperature alters interactions and keystone taxa in the marine microbiome.

Ewa Merz[1]*, Riley Hale[1], Erik Saberski[1], Kasia M. Kenitz[1,2], Melissa Carter[1,2], Jeff S. Bowman[1], Andrew D. Barton[1,3]

[1]Scripps Institution of Oceanography, University of California San Diego, La Jolla, CA 92037, United States

[2]Southern California Coastal Ocean Observing System, University of California San Diego, La Jolla, CA 92037, United States

[3]Department of Ecology, Behavior and Evolution, University of California San Diego, La Jolla, CA 92039, United States

*Corresponding author: Ewa Merz.

Email: e2merz@ucsd.edu

Contents

Supplementary Materials and Methods …………………………………….. Pages 4-9

Calculate the Euclidean distance between sampling points

Parametrize the local regularized regression

Extract the local model coefficients

Differences from the original MDR S-map approach

Interpretation of the inferred interaction strengths

Limitations of using relative abundance data

Increase confidence in inferred interaction strengths

Effect of seasonality on inferred interaction strengths

References

Supplementary Figures ………………………………………………………. Pages 10-23

Fig. S1. The effect of methodological simplifications and relative abundance data on the accuracy of inferred interaction strengths based on the MDR S-map's coefficients.

Fig. S2. Example of changing interaction coefficients for a microbial ASV from the Methanomassilicoccales (Archaea) group.

Fig. S3. Estimating the ideal bin size to create equally spaced sampling intervals.

Fig. S4. The effect of seasonality in estimating the MDR S-map coefficients as a proxy of interaction strength.

Fig. S5. Pairwise interaction matrix between most frequent microbial ASVs.

Fig. S6. Correlation between interaction metrics and the relative abundance of microbial ASVs.

Fig. S7. Rank abundance curve for microbial ASVs.

Fig S8. NMDS analysis of non-keystone microorganisms.

Fig. S9. Distance between points in NMDS.

Fig. S10. Temperature dependency of interactiveness of all keystone taxa.

Fig. S11. Temperature dependency of percent facilitation of selected keystone taxa.

Fig. S12. Correlation between relative abundance and interactiveness of keystone microorganisms as a function of water temperature.

Fig. S13. Seasonal succession of keystone microorganisms.

Fig. S14. Test statistics for microbial interaction across a water temperature gradient.

Supplementary Materials and Methods

Calculate the Euclidean distance between sampling points

Nonlinearity and chaos are common in natural community dynamics [1, 2]. MDR S-maps accommodate nonlinear dynamics by giving data points closer in state space a higher weight. The number of data points required to reconstruct a state space increases exponentially with the number of variables (coordinate axes). We overcome this limitation by averaging the distance between data points in multiple low-dimensional state-space reconstructions [3, 4].

We used the target ASV and nine other randomly selected ASVs (embedding dimension E = 10) to generate low-dimensional state-space reconstructions (embeddings). Because of computational limitations, we randomly generated only 1000 embeddings. We estimated the two-timesteps-ahead (tp = 2) forecast performance of those embeddings using multivariate simplex projection on our target taxa. We used the first half of the time series to reconstruct the attractor and the other half to generate predictions [5]. We chose tp = 2 to avoid temporal autocorrelation and to maintain a meaningful time frame for observing biological interactions in our system (8 days). The forecast performance was calculated by performance $= ((RMSE_{max} - RMSE \; + RMSE_{min})^2 + \rho^2)^{0.5}$. We chose this metric because it includes both forecast skill (ρ, Pearson's correlation between observations and predictions) and forecast error (root-mean-squared error, or RMSE). Lower RMSE indicates better forecast performance. Therefore, we inverted the RMSE before calculating the overall performance. We then kept the top 100 embeddings with the highest performance.

We calculated the Euclidean distance among points in the top 100 embeddings. Then, we averaged distances among points across all 100 embeddings, giving higher weight to those with higher predictive performance. The resulting metric, called the multiview distance, was used to fit local regularized regressions with many variables.

Parametrize the local regularized regression

We used the coefficients of a local elastic-net regression to approximate the time-varying interaction strengths [3, 6, 7]. Local in this context means that the data used to fit the regression are weighted differently depending on their proximity in state-space (defined by the previously estimated multiview distance). Closer points in the state-space were assigned greater weight during model fitting. The weight was obtained by fitting an exponential decay function to the multiview distance calculated in the previous section [3]. The decay function's exponent was multiplied by the parameter Θ, which controled the regression's degree of nonlinearity (high Θ = nonlinear; small or zero Θ = linear). Regularization techniques in the fitting process allowed the regression to generalize, cope with observational noise, and improve its performance on unseen data (i.e., prevent under- and overfitting). In regularization, large coefficients are penalized by adding a

penalty term to the loss function, discouraging the regression from becoming too complex. Two types of penalties exist, called lasso and ridge. The lasso penalty adds the absolute value of the regression coefficients to the loss function, whereas the ridge adds the squared coefficients. In both, the parameter λ controls the penalty's severity (large λ = strong penalty, pushes most coefficients towards or close to 0). Elastic-net regression uses a mixture of lasso and ridge penalty. The parameter α controls the balance between the lasso and ridge penalty used in elastic-net regression (if α = 0.5, we use an equal mix of both, whereas with α = 1 we exclusively use lasso penalty and α = 0 ridge penalty).

Before fitting and extracting coefficients, we had to parametrize the local elastic-net regressions by finding the best values for Θ, λ, and α that maximized their performance using leave-future-out cross-validation [3, 7]. We used all possible combinations (n=125) of Θ (0, 0.1, 1, 3, 8), α (0.1, 0.3, 0.5, 0.7, 0.9), and λ (1.000, 0.178, 0.032, 0.006, 0.001) to test how well the predictions made by the MDR S-map match the observed data. Leave-one-out cross-validation, where iteratively one value is left out and the rest of the data is used to predict this value, is a widely used technique to test a model's performance [8]. However, explicit training and testing data sets are more robust than leave-one-out cross-validation, especially for long time series data [9]. Moreover, time series have temporal dependencies that must be preserved (i.e., we should not use future points to predict past values). Therefore, we parametrized the MDR S-maps using "leave-future-out" to overcome this issue. The initial library contains only the first 10% (n = 49) data points, which are used to predict the next point (here, t =50). This point is then added to the library (n = 50) to predict the next point (here, t = 51). With this approach, we predicted the remaining 90% of the data, constantly increasing our library size with the predicted value. We evaluated the predictive performance of the MDR S-maps, analogous to that of the multivariate simplex projection, with performance $= ((RMSE_{max} - RMSE \ + RMSE_{min})^2 + \rho^2)^{0.5}$.

Extract the local model coefficients

We discarded all parameter combinations that did not outperform a persistence model regarding forecast error (RMSE) and skill (ρ). A persistence model assumes that there is no change between the current and predicted values. Among the remaining combinations, many exhibited similar forecast performance but varied significantly in estimating interaction strengths. Therefore, we took a weighted average of the coefficients from the best-performing combinations, which fall within the upper 95% quantile, to assess interactions with greater accuracy [10]. Additionally, the standard deviation of the coefficient values and their occurrence across combinations allowed us to evaluate their variability and uncertainty. For 17 ASVs, we were unable to calculate their interaction strengths because none of the parameter combinations outperformed the persistence model, or the skill (ρ) was smaller than 0.2. As a result, we excluded those ASVs from further analysis.

Differences from the original MDR S-map approach

We simplified the original MDR S-map approach proposed by a previously published paper to make it conceptually easier and computationally less intensive, and maintain the same efficacy in inferring interaction strengths [3]. In detail, we:

1. We set E = 10 for all ASVs. We did not estimate the optimal E value for each ASV using univariate simplex projection, as many possible E values yielded very similar predictive performance in our case.
2. We included all ASVs as variables for the multivariate embeddings, disregarding the possibility that they might not be causally linked (tested using convergent cross-mapping). This pre-selection of interactions is computationally intensive and only applies to the MDR distance calculations; for the final S-map models, even the original approach used all taxa—independent if they significantly cross-mapped.
3. Instead of 10,000 random multivariate embeddings, we selected the top embeddings out of 1,000 to reduce computation time.
4. We selected the top 100 multivariate embeddings to estimate the multiview distance based on a combination of forecast error and skill, rather than just considering skill.
5. We used leave-future-out cross-validation instead of leave-one-out cross-validation for the MDR S-map parametrization and extracting the local model coefficients, as this approach was more suitable for long-term time series data.
6. Instead of 2,025 parameter combinations, we parametrized the MDR S-map using only 125 parameter combinations of α, Θ, and λ. With more than 125 parameter combinations, inferring interaction strengths did not significantly improve, and the computational resources needed were drastically reduced.
7. We discarded parameter combinations that did not outperform the constant predictor in forecast skill (ρ) and error. Then, we estimated the model coefficients from the best-performing remaining combinations and calculated a weighted average based on forecast performance.

To ensure that our simplified approach did not reduce the MDR S-map efficacy in estimating interactions, we compared theoretical and estimated interaction strengths from the same theoretical population model (Multispecies Ricker Model) that has been used to validate the original approach [3].

We found that the simplified approach performed as well as the original approach (**Fig. S1A-B**) in inferring interactions with other taxa. The correlation coefficient between the theoretical and inferred Jacobian coefficients by the MDR S-map was around 0.9 for both the original and simplified approaches. Whereas the simplified approach detected more true positive and negative interactions (**Fig. S1C-D**), the original approach detected more true zeros and fewer false positive or negative interactions (**Fig. S1E**). This might have resulted from the model averaging in the simplified approach, making it more likely to detect an interaction. Both original and simplified approaches did not detect many wrong

signs, i.e., positive interactions were recorded as negative ones or vice versa (max. 1.5%, **Fig. S1F**).

Interpretation of the inferred interaction strengths

The coefficients obtained from the MDR S-map served as an approximation of the Jacobian interaction matrix. The elements of the Jacobian indicated the strength of interaction or the net local effect that each taxon in the community (including itself) has on the predicted taxon [6]. It is important to distinguish between the Jacobian elements and the per capita interaction coefficients. The MDR S-map does not recover the interaction coefficients, which represent the per capita effect one taxon has on the growth rate of another taxon [11]. To obtain the per capita interaction coefficients, one would need to predict the growth rate of the target taxon rather than its abundance [12].

We would like to clarify the interpretation of estimated interaction strengths with an example. Suppose we aim to study the effect of microorganism B on microorganism A (microorganism B → microorganism A). The MDR S-map estimates a coefficient of 0.01 for microorganism B. This coefficient means that if the abundance of microorganism B increases by 1 unit, the abundance of microorganism A will increase by 0.01 units, assuming that the abundance of all other microorganisms (including A) remains constant.

The coefficients were estimated based on scaled data, so we cannot directly interpret them as changes in relative abundance. Additionally, we anticipate that the magnitude of the coefficients may be underestimated. This underestimation can occur due to the distribution of variance in the predictor variable across many explanatory variables (n = 184), the penalization of large coefficients in regularized regression, and the averaging over multiple models (see also **Fig. S1**).

Limitations of using relative abundance data

The constant sum nature of percentage data introduces noise into the MDR S-map coefficients, potentially leading to either overestimation or underestimation of ASV abundances. Moreover, the relative abundances of ASVs are not independent; for instance, an increase in the abundance of two ASVs may occur simply because another ASV's abundance has decreased. The introduced noise and interdependence can hinder our ability to accurately estimate the true coefficients (as discussed in the analysis by a previous study [3] and illustrated in **Fig. S1**). Unfortunately, obtaining absolute abundance data for microbial communities is challenging, especially when differentiating between ecotypes [13]. Validations using the Multispecies Ricker Model show that we can still identify underlying interactions, albeit with reduced accuracy compared to when using absolute abundance data (**Fig. S1**) [3].

Increase confidence in inferred interaction strengths

We took several steps to increase confidence in our inferred interaction strengths. In detail, we:

1. Include a strong environmental driver (i.e., surface temperature) to control for external factors such as co-occurring seasonal changes in abundance.
2. Consider many models (parameter combinations) to infer MDR S-map coefficients and estimate their uncertainty.
3. Only use dominant ASVs whose time series are well resolved (occurrence > 50% and mean relative abundance > 0.1%)
4. Only use ASVs that we can predict with a skill greater than 0.2 and a performance greater than the constant predictor.
5. Only keep strong interactions (occurrence over time and strength lie above the 50% quantile)

Effect of seasonality on inferred interaction strengths

Microbial taxa may appear to interact if their abundances follow similar seasonal patterns. To ensure that seasonal signals did not influence our inferred interaction strengths, we took two steps: i) we removed the seasonal signal from the time series, and ii) we created seasonal surrogates and repeated the MDR S-map analysis described above, similarly to how we analyzed the original time series.

To remove the seasonal signal, we decomposed the time series using the `decompose` function from the R package `stats` (version 4.4.0) and kept the seasonal part. To create seasonal surrogates, we reshuffled the time series while preserving the monthly seasonal signal. In simpler terms, we grouped the time series by month and rearranged the data points within each month (for example, all data points collected in January). We only transformed the time series for the non-target variables, while keeping the target variable at the current time point and the next time point in their original form. In **Fig. S4A**, we show the original and transformed time series for water temperature.

Removing the seasonal signal did not affect the number of detected links, net interaction strength, or interactiveness (**see Fig. S4B-D**). However, when we used seasonal surrogates instead of the original time series, we discovered many fewer links. Specifically, we found a median of 7 links compared to 31 in the original time series. Additionally, both net interaction strength and interactiveness were much lower. The net interactiveness decreased from a median of 0.12 to 0.02 when using seasonal surrogates. Similarly, interactiveness fell from a median of 0.58 to 0.06. This indicates that the interactions inferred by the MDR S-map are not solely a result of seasonal co-occurrence patterns.

References

1. Clark TJ, Luis AD. Nonlinear population dynamics are ubiquitous in animals. *Nature Ecology & Evolution* 2019; **4**: 75–81. doi:10.1038/s41559-019-1052-6

2. Munch S, Rogers TL, Johnson BJ, Bhat U, Tsai C. Rethinking the prevalence and relevance of chaos in ecology. *Annu Rev Ecol Evol Syst* 2022, **53**: 227-249. doi:10.1146/annurev-ecolsys-111320-052920

3. Chang C-W, Miki T, Ushio M, Ke P-J, Lu H-P, Shiah F-K, et al. Reconstructing large interaction networks from empirical time series data. *Ecol Lett* 2021; **24**: 2763–2774. doi:10.1111/ele.13897

4. Ye H, Sugihara G. Information leverage in interconnected ecosystems: Overcoming the curse of dimensionality. *Science* 2016; **353**: 922–925. doi:10.1126/science.aag0863

5. Sugihara G, May RM. Nonlinear forecasting as a way of distinguishing chaos from measurement error in time series. *Nature* 1990; **344**: 734–741. doi:10.1038/344734a0

6. Deyle ER, May RM, Munch SB, Sugihara G. Tracking and forecasting ecosystem interactions in real time. *Proc Biol Sci* 2016; **283**: 20152258. doi:10.1098/rspb.2015.2258

7. Cenci S, Sugihara G, Saavedra S. Regularized S‐map for inference and forecasting with noisy ecological time series. *Methods Ecol Evol* 2019; **10**: 650–660. doi:10.1111/2041-210X.13150

8. Yates LA, Aandahl Z, Richards SA, Brook BW. Cross validation for model selection: A review with examples from ecology. *Ecol Monogr* 2023; **93**: e1557. doi:10.1002/ecm.1557

9. Munch SB, Rogers TL, Sugihara G. Recent developments in empirical dynamic modelling. *Methods Ecol Evol* 2023; **14**: 732–745. doi:10.1111/2041-210X.13983

10. Cenci S, Saavedra S. Uncertainty quantification of the effects of biotic interactions on community dynamics from nonlinear time-series data. *J R Soc Interface* 2018; **15**: 20180695. doi:10.1098/rsif.2018.0695

11. Arditi R, Tyutyunov YV, Titova LI, Rohr RP, Bersier L-F. The Dimensions and Units of the Population Interaction Coefficients. *Frontiers in Ecology and Evolution* 2021; **9**: 775754. doi:10.3389/fevo.2021.775754

12. Nguyen PL, Pomati F, Rohr RP. Inferring coexistence likelihood in changing environments from ecological time series. *Proc Natl Acad Sci U S A* 2025; **122**: e2417905122. doi:10.1073/pnas.2417905122

13. Röttjers L, Faust K. From hairballs to hypotheses-biological insights from microbial networks. *FEMS Microbiol Rev* 2018; **42**: 761–780. doi: 10.1093/femsre/fuy030

Supplementary Figures

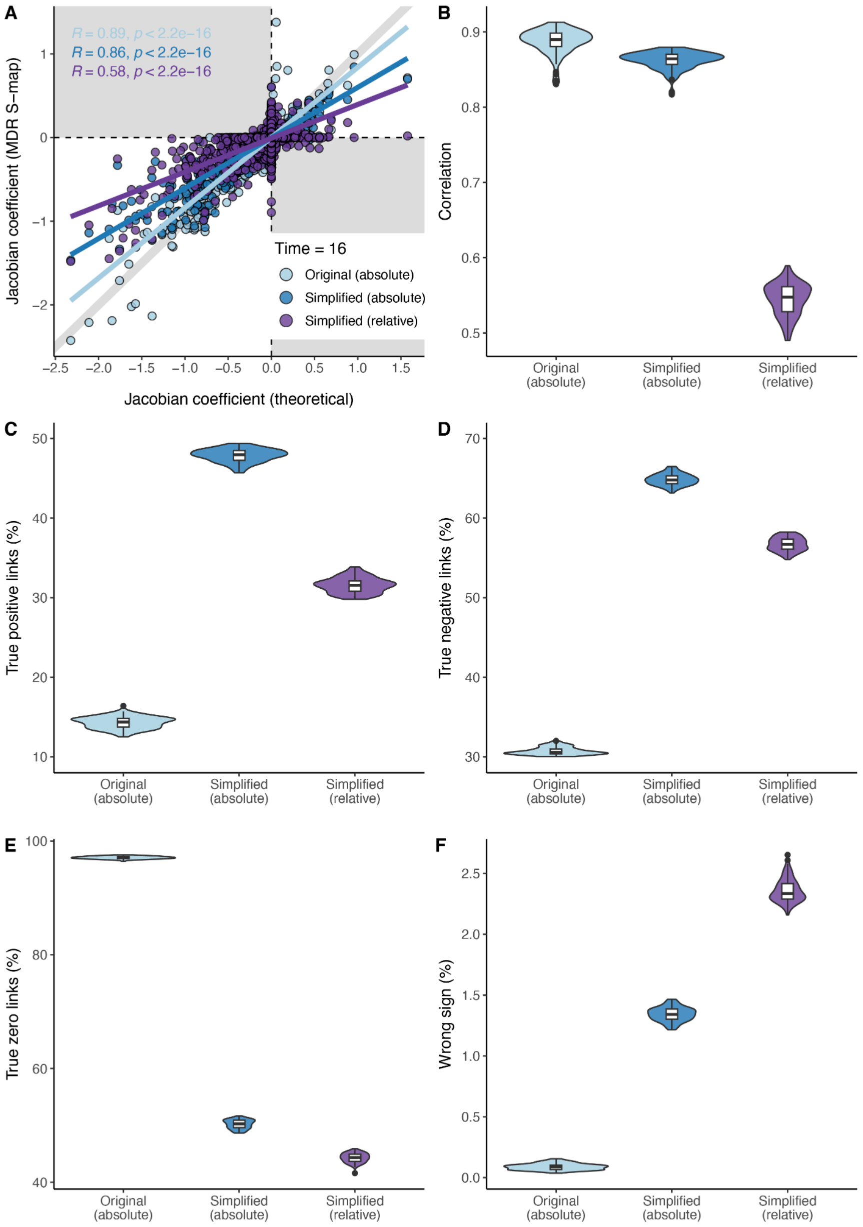

Fig. S1. The effect of methodological simplifications and relative abundance data on the accuracy of inferred interaction strengths based on the MDR S-map's coefficients. Comparing interaction coefficients from the Multispecies-Ricker Model between theoretical expectation (specified in the model) and those based on coefficients estimated by the MDR S-map. (A) Correlation between estimated and theoretical interaction coefficients for a single time point. (B) Correlation across all time points. (C) Percentage of detected positive links. (D) Percentage of detected negative links. (E) Percentage of detected non-interactions (interaction coefficient should be zero). (F) Interactions with the wrong sign (i.e., positive interaction instead of a negative one or vice versa).

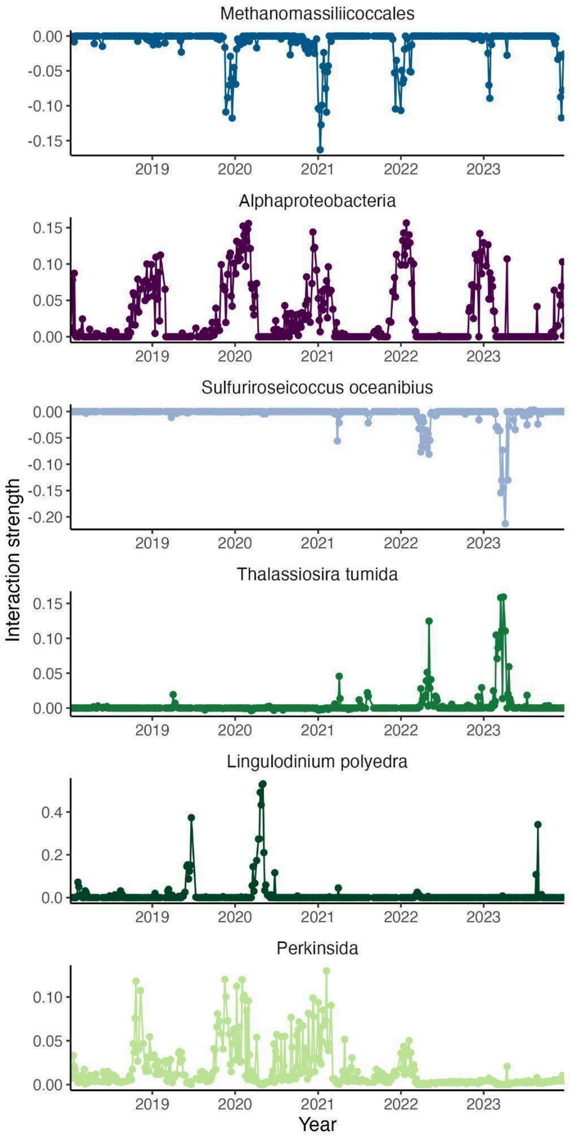

Fig. S2. Example of changing interaction coefficients for a microbial ASV from the Methanomassilicoccales (Archaea) group. The y-axis shows the changing interaction strength of a pairwise interaction estimated by the coefficient of the MVD S-map. Colors of dots and lines correspond to higher taxonomic groups shown in **Fig. 1A**.

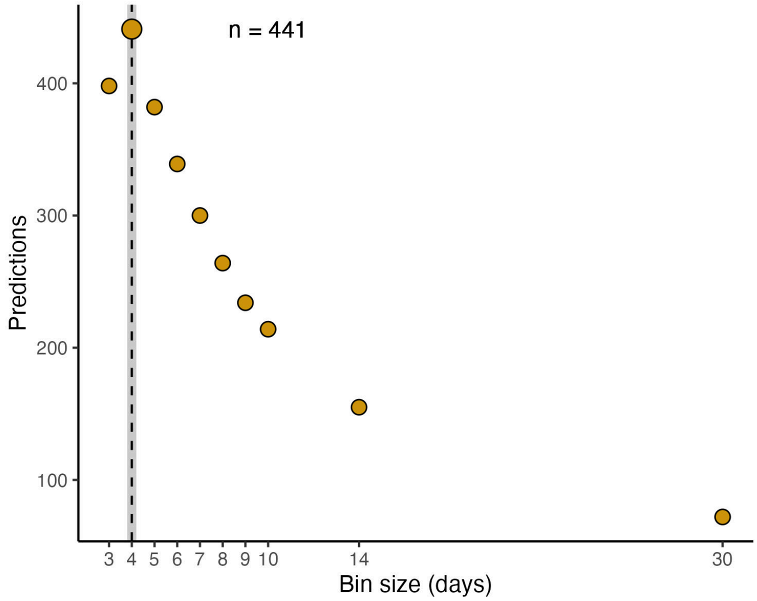

Fig. S3. Estimating the ideal bin size to create equally spaced sampling intervals. The dashed line shows the ideal bin size, which maximizes the number of possible predictions (data points) for the analysis. N stands for the number of data points at the ideal bin size.

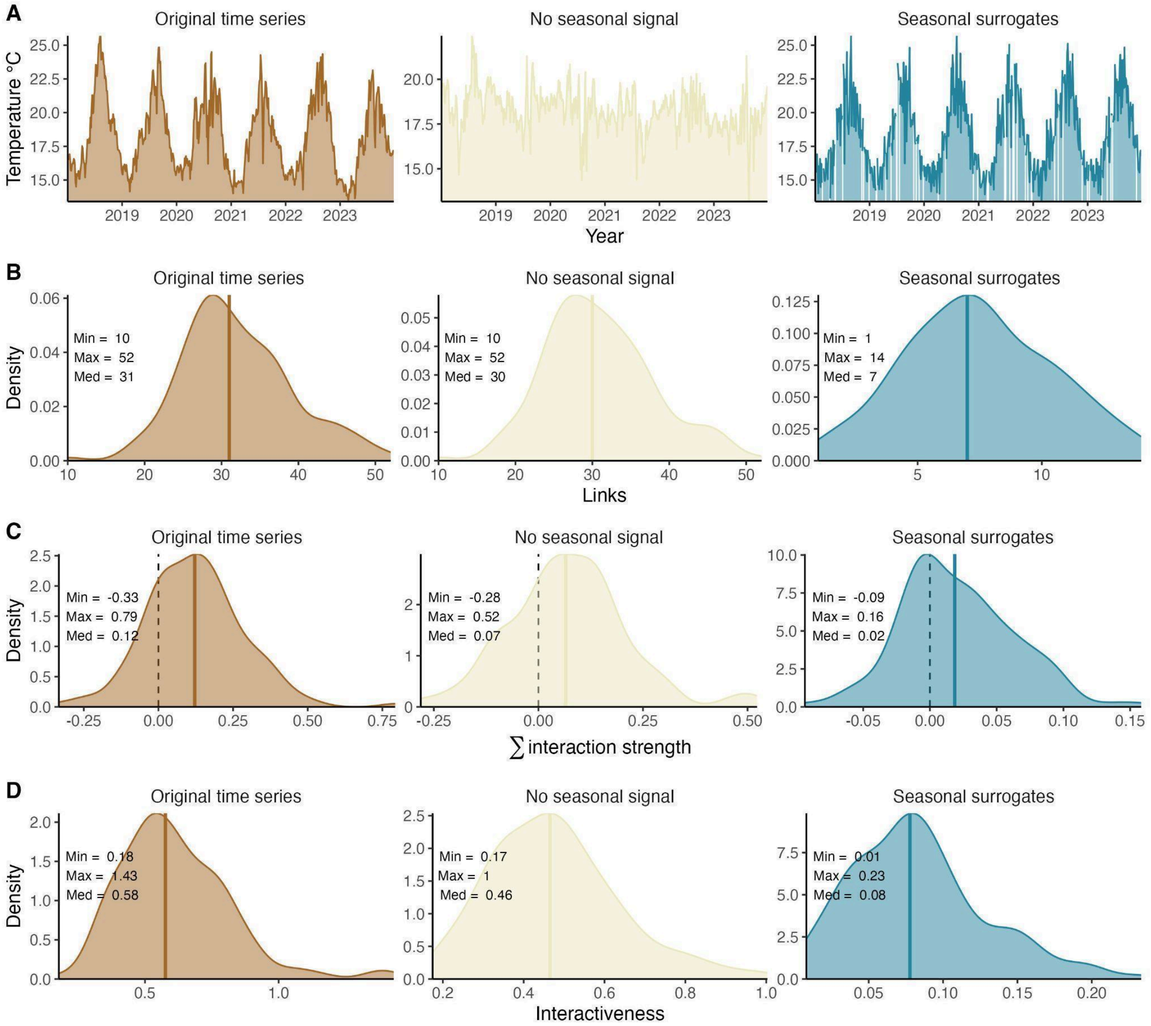

Fig. S4. The effect of seasonality in estimating the MDR S-map coefficients as a proxy of interaction strength. (A) Here is an example of how the time series transformation appears for water temperature measured in °C. On the left, you will see the original time series. In the middle, the time series with the seasonal signal removed can be observed. Finally, on the right, the seasonal surrogates are displayed, which were created by reshuffling the time series while preserving the seasonal signal. In (B-D), we compare the MDR S-map results for the original time series (left), the time series with the removed seasonal signal (middle), and the seasonal surrogate times series (right) for (B) the number of detected links (MDR S-map coefficients different from 0), (C) the net interaction strength and (D) interactiveness. The solid line shows the median of the distribution. The dashed line in (C) shows where the net interaction strength is zero. Min stands for minimum, Max for maximum, and Med for median of the density distribution.

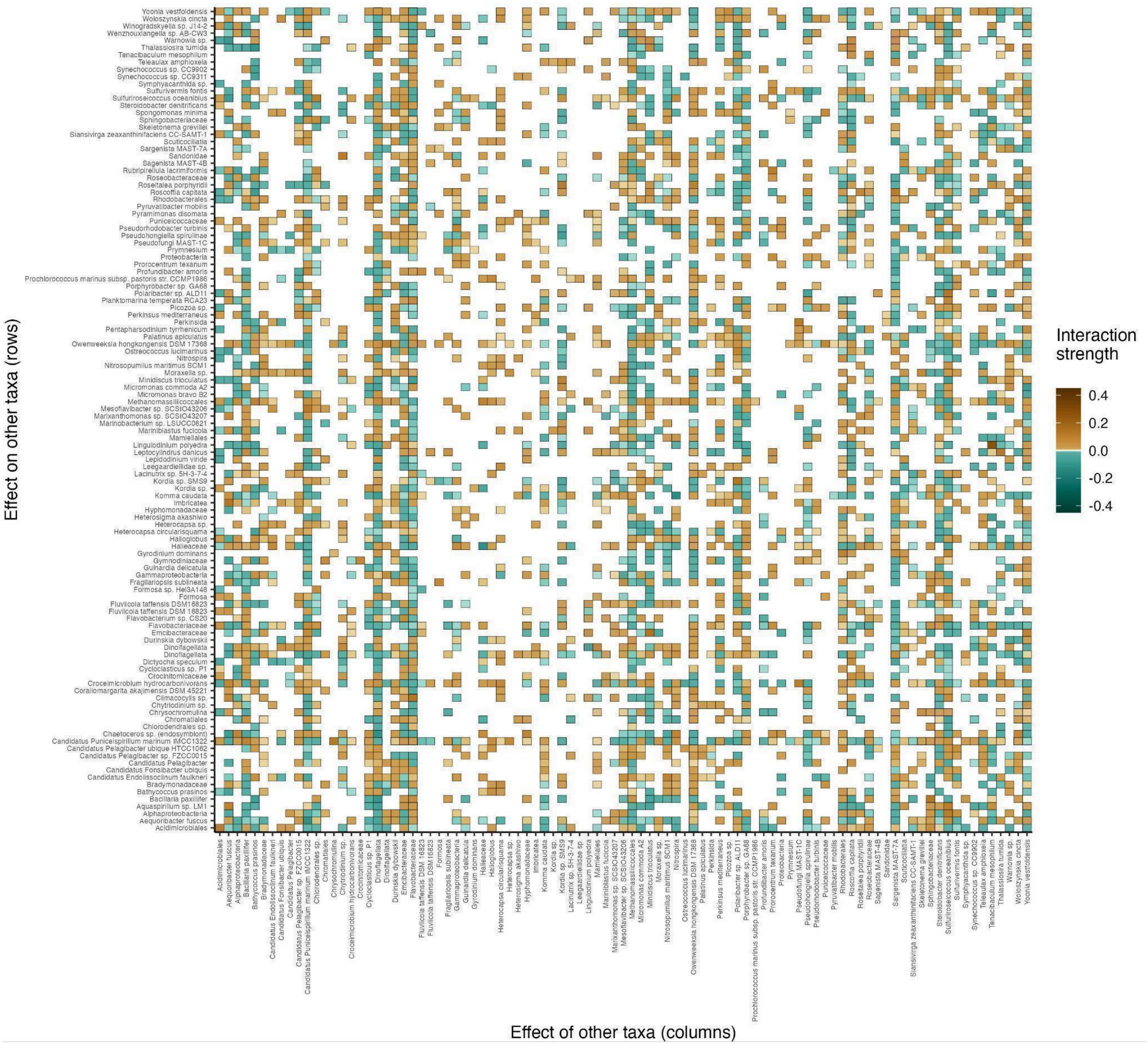

Fig. S5. Pairwise interaction matrix between most frequent microbial ASVs. ASVs are ordered alphabetically. Filled squares show the average interactions over time. Examining the rows reveals how different taxa influence those shown in the column of the interaction matrix. Colors correspond to the strength of an interaction, where green means negative and brown means positive interaction.

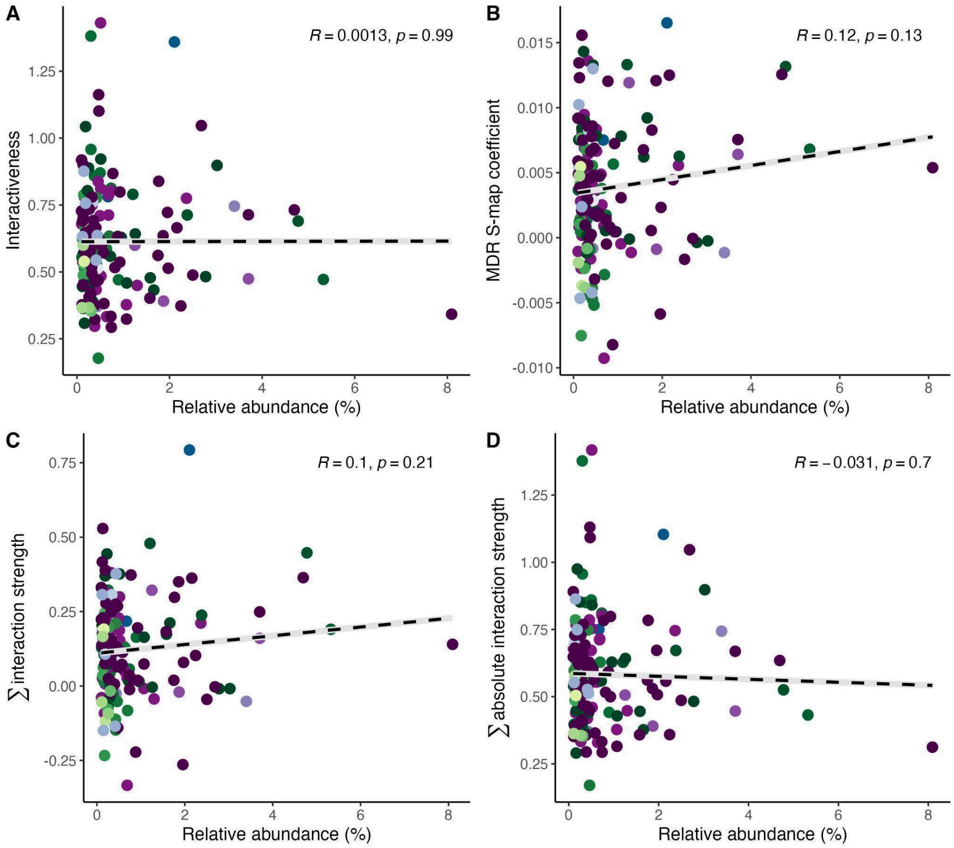

Fig. S6. Correlation between interaction metrics and the relative abundance of microbial ASVs. Points show the average over time. Dashed lines show non-significant relationships (P-value > 0.05), while solid lines show significant relationships (P-value < 0.05). R stands for the correlation coefficient (slope of the regression line), and p for the P-value. Colors of dots and correspond to higher taxonomic groups shown in **Fig. 1A**.

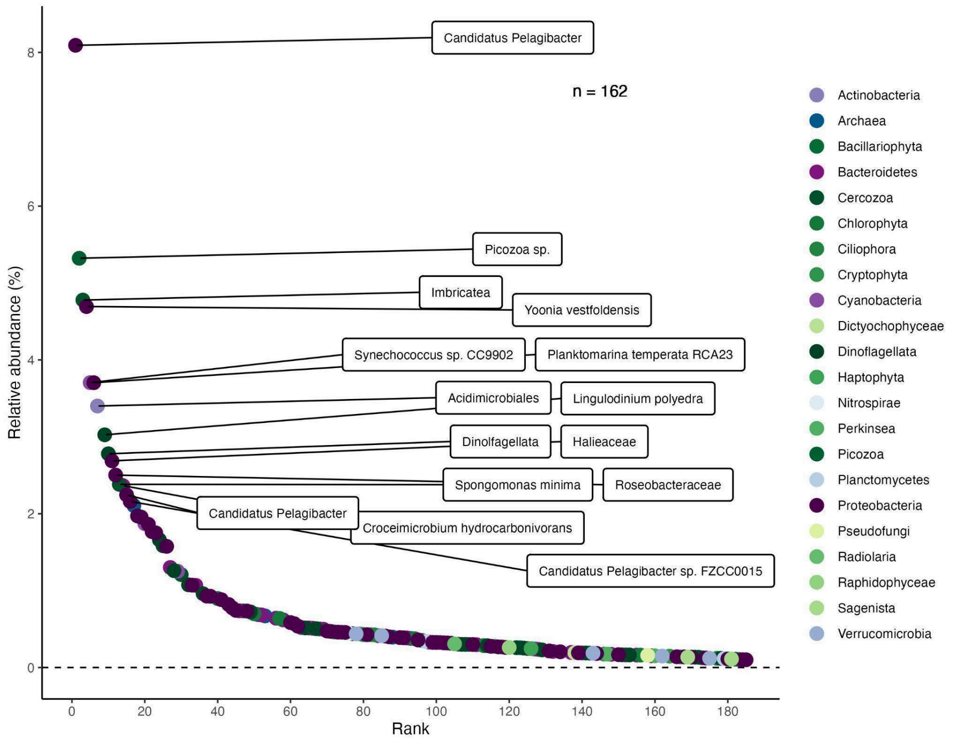

Fig. S7. Rank abundance curve for microbial ASVs. The names of the most common ASVs are displayed. Dots show the mean relative abundance over time. Colors of dots correspond to higher taxonomic groups shown in the figure legend and in **Fig. 1A**. N represents the number of points (ASVs) shown in the plot.

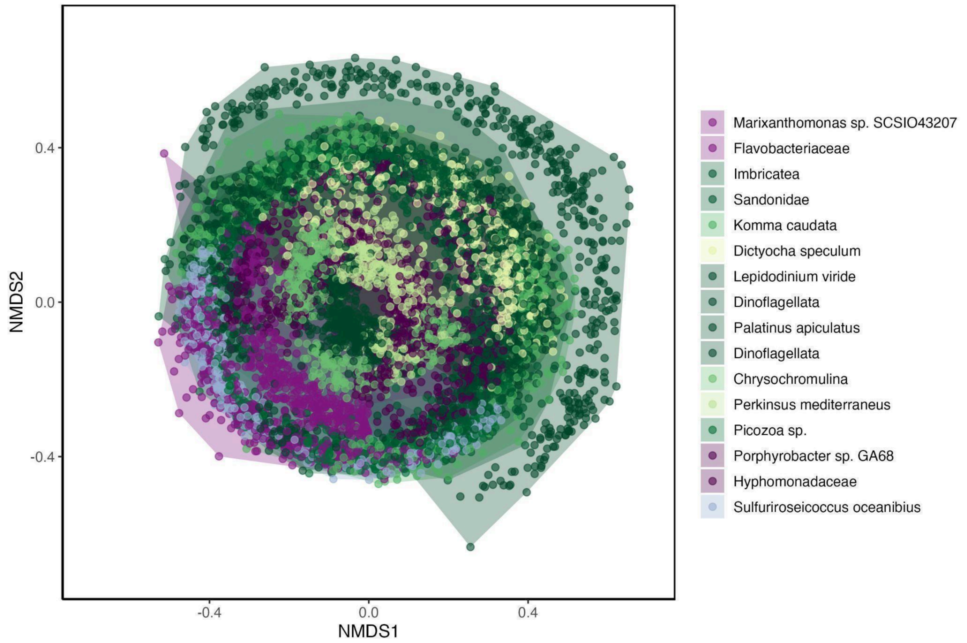

Fig S8. NMDS analysis of non-keystone microorganisms. Dots display a single time point, while shapes are polygons containing all points of a given keystone microorganism. Non-keystone microorganisms were selected randomly from the whole community. Points of non-keystone taxa are farther away than keystone taxa highlighted in **Fig 3B**, indicating a higher level of consistency of keystone taxa. Colors of dots and polygons correspond to higher taxonomic groups shown in **Fig. 1A**. We used a stress threshold < 0.3 because none of the observations had a stress value lower than 0.2

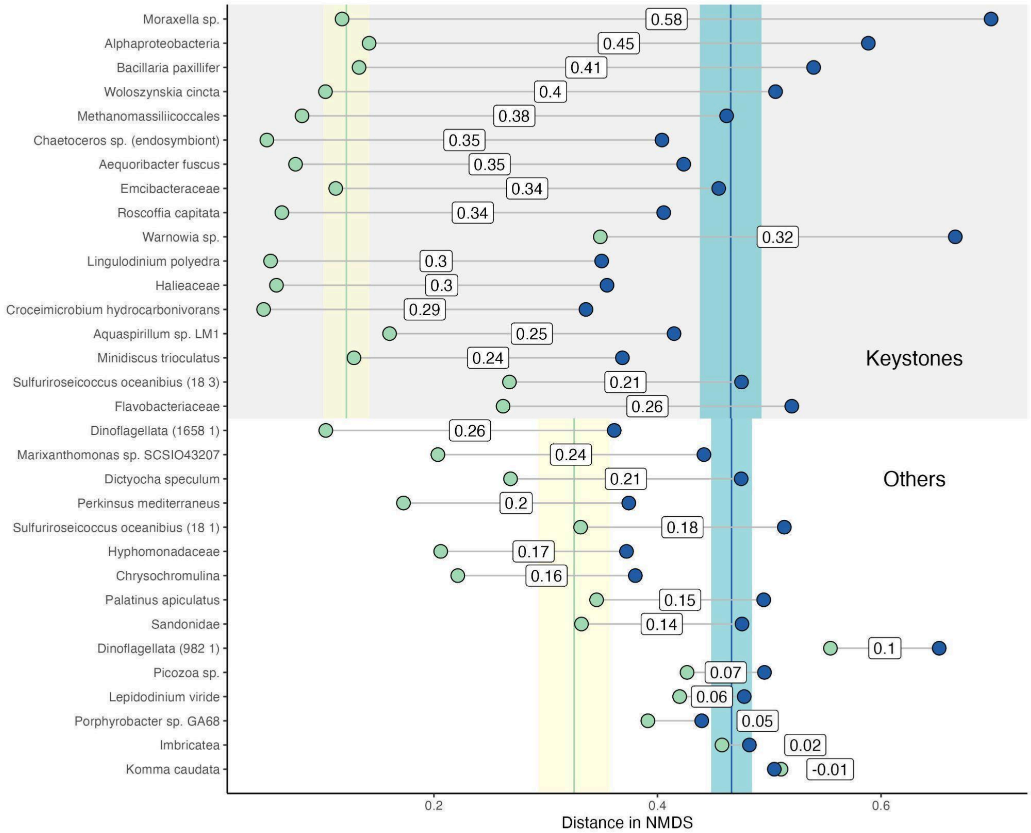

Fig. S9. Distance between points in NMDS. Green points show the distance within a given taxa, whereas blue points show the distance between different taxa. Numbers show the difference between distances within a taxon and between taxa. The bars show the mean, and the shading shows the standard error of the mean.

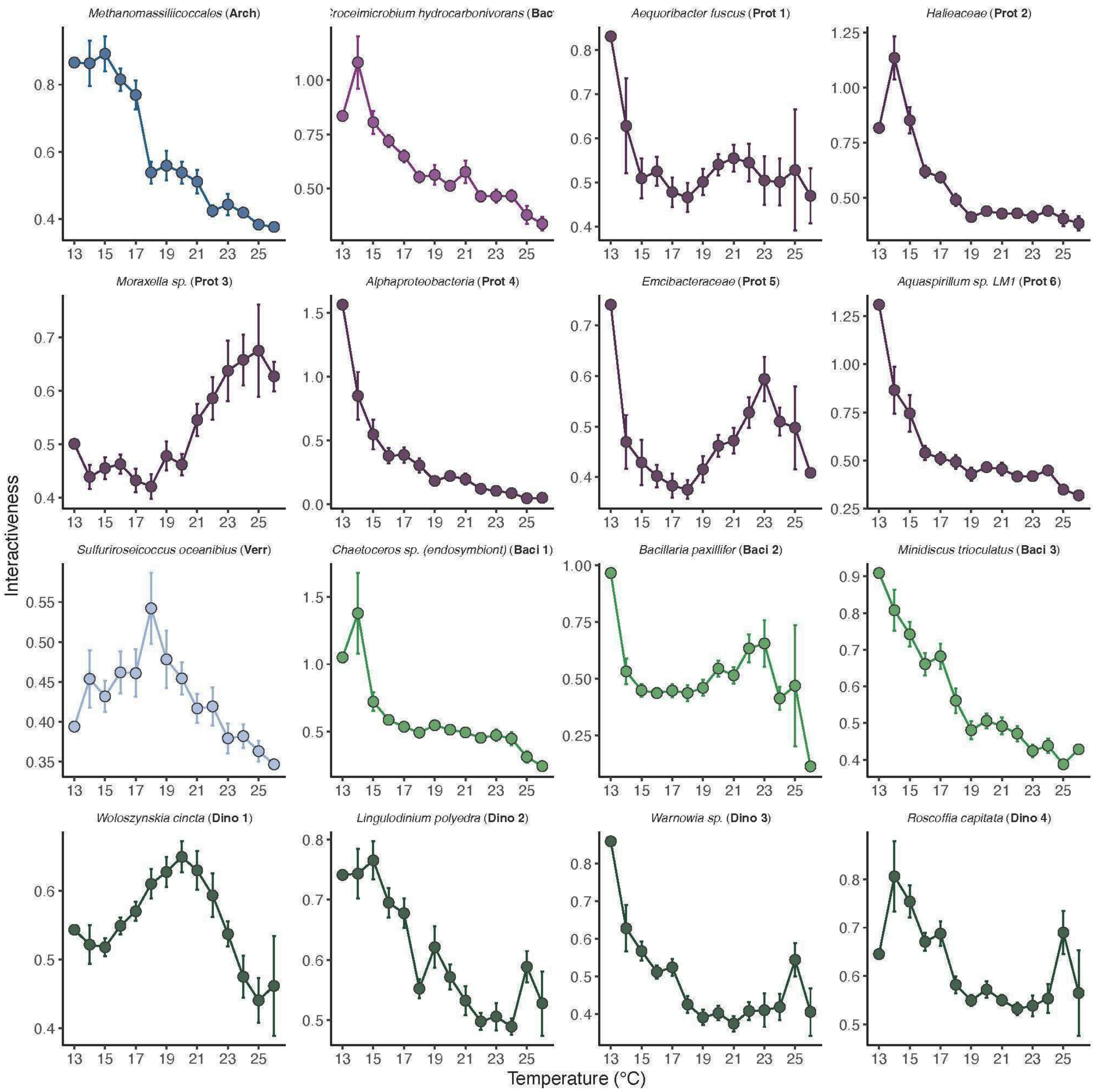

Fig. S10. Temperature dependency of interactiveness of all keystone taxa. This plot is an extended version of **Fig. 4A**. Error bars show the standard error of the mean (SE) using all interaction data from a given temperature. Colors of dots and lines correspond to higher taxonomic groups shown in **Fig. 1A**.

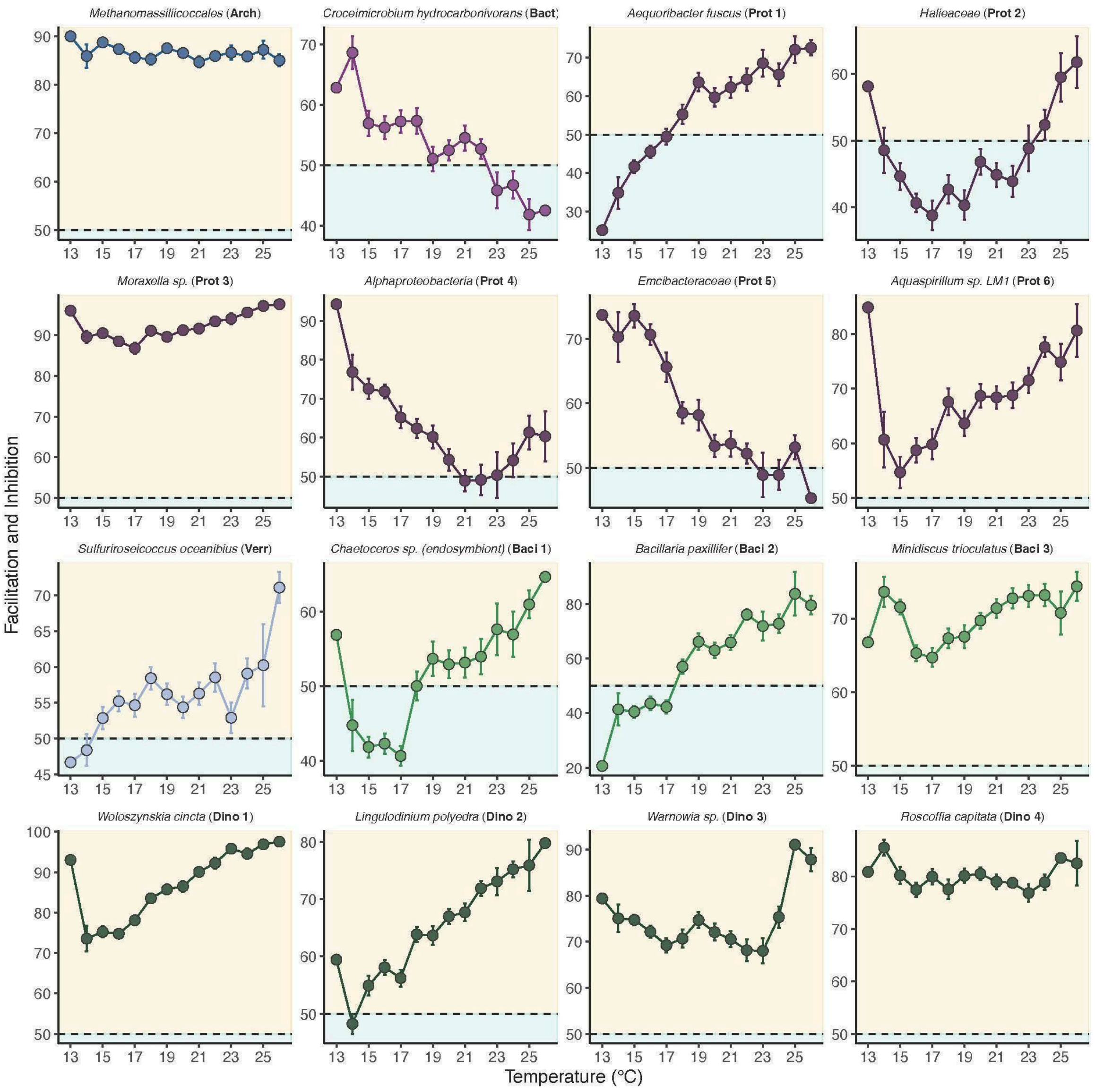

Fig. S11. Temperature dependency of percent facilitation of selected keystone taxa. This plot is an extended version of **Fig. 4B**. Percent facilitation is calculated by dividing the number of positive interactions by the total number of interactions at a given temperature. The shading denotes the net positive (facilitation > 50%) versus net negative interactions (facilitation < 50%). Error bars show the standard error of the mean (SE) using all interaction data from a given temperature. Colors of dots and lines correspond to higher taxonomic groups shown in **Fig. 1A**.

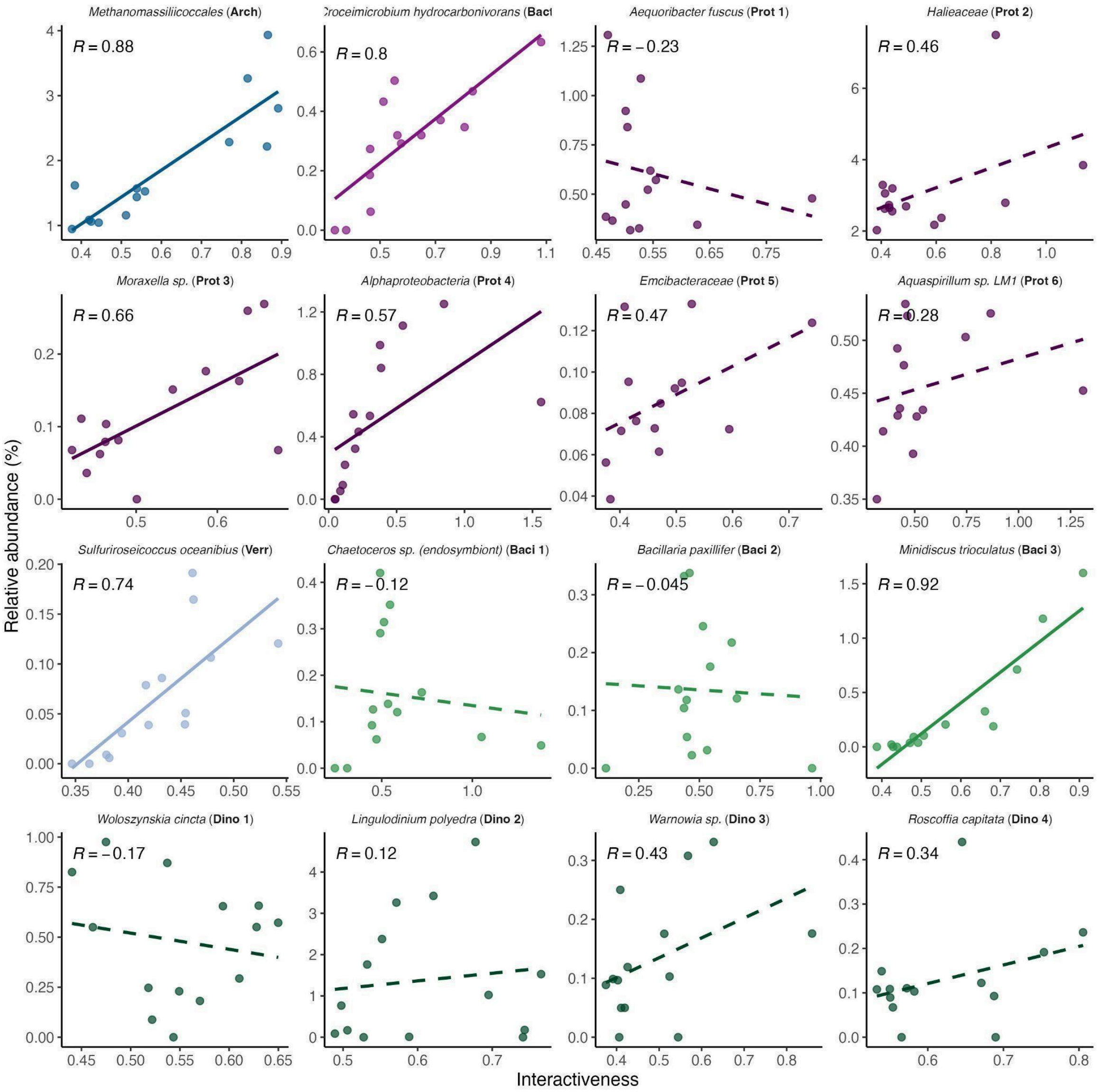

Fig. S12. Correlation between relative abundance and interactiveness of keystone microorganisms as a function of water temperature. Points show the average for a given temperature level. Dashed lines show non-significant relationships (P-value > 0.05), while solid lines show significant relationships (P-value < 0.05). R stands for the correlation coefficient (slope of the regression line). Colors of dots and lines correspond to higher taxonomic groups shown in **Fig. 1A**.

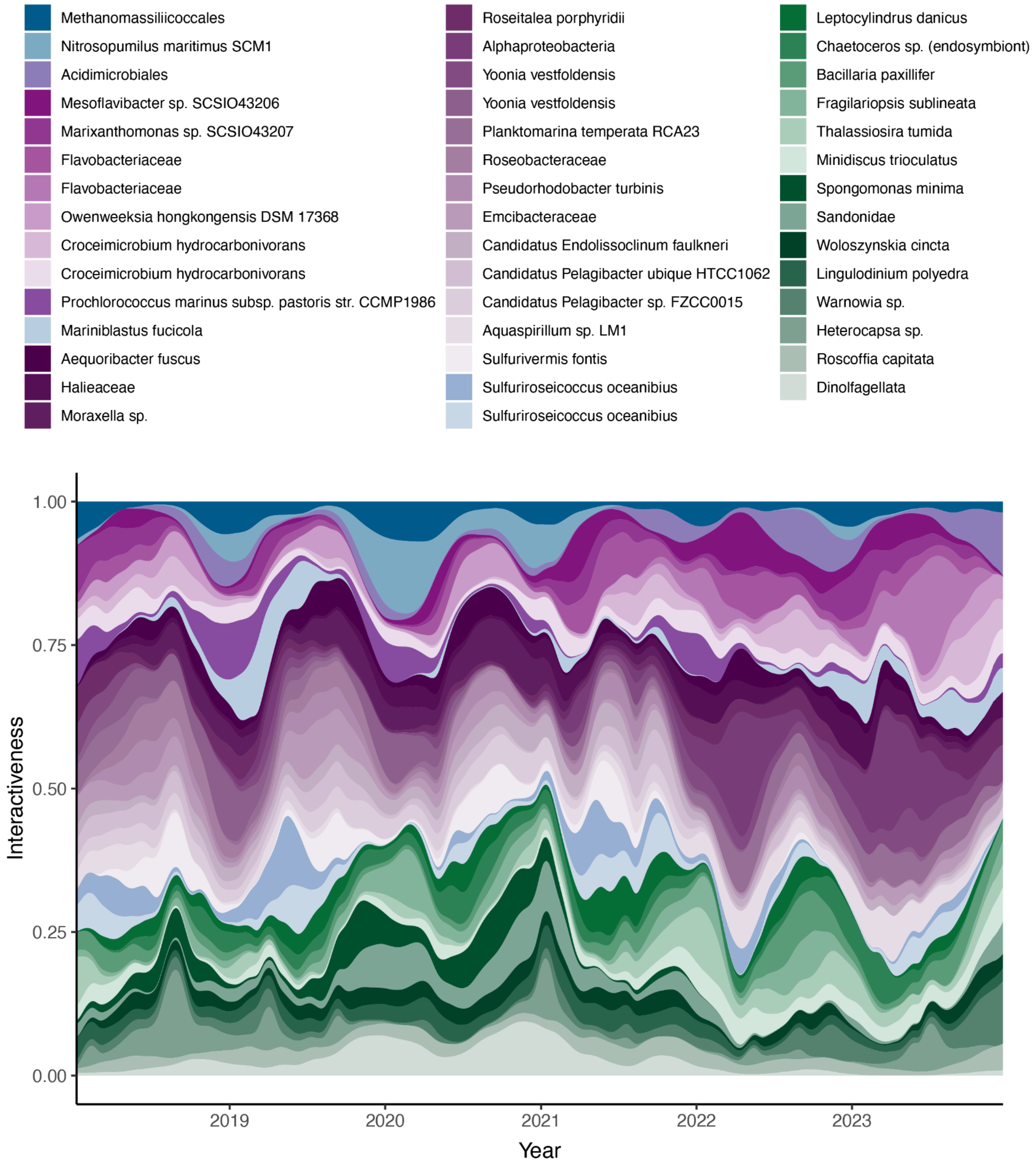

Fig. S13. Seasonal succession of keystone microorganisms. Interactiveness (y-axis) was normalized between 1 and 0 for each sampling day. To reduce complexity, only microorganisms from **Fig. 4C** are displayed. Colors correspond to higher taxonomic groups shown in **Fig. 1A**.

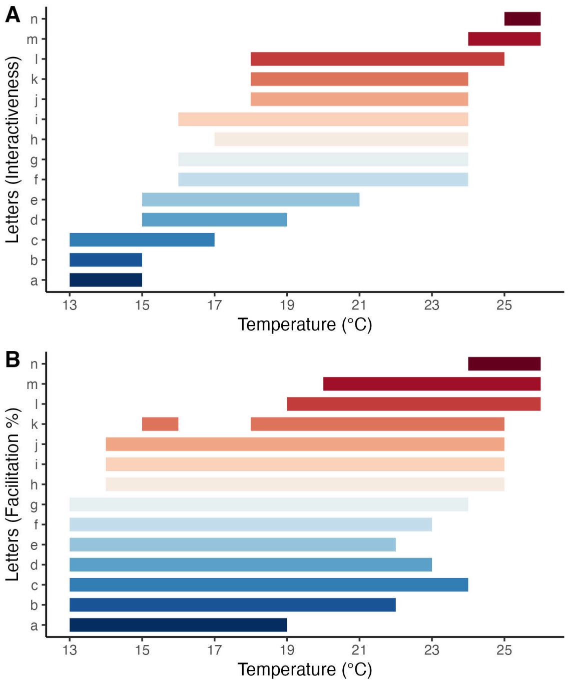

Fig. S14. Test statistics for microbial interaction across a water temperature gradient. (A) Interactiveness and (B) Percentage of facilitative (positive) interactions when compared to negative ones. This Figure helps interpret the statistical significance of **Figure 4D-E**.
